# CardioVinci: building blocks for virtual cardiac cells using deep learning

**DOI:** 10.1098/rstb.2021.0469

**Published:** 2022-11-21

**Authors:** Afshin Khadangi, Thomas Boudier, Eric Hanssen, Vijay Rajagopal

**Affiliations:** ^1^ Department of Biomedical Engineering, Faculty of Engineering and Information Technology, University of Melbourne, Parkville, Australia; ^2^ Institut de Biologie Paris-Seine, Sorbonne Université Campus Pierre et Marie Curie, Paris, France; ^3^ Ian Holmes Imaging Center, Bio21, University of Melbourne, Parkville, Victoria, Australia

**Keywords:** cell architecture, generative adversarial networks, electron microscopy, cardiac cell, three-dimensional model

## Abstract

Advances in electron microscopy (EM) such as electron tomography and focused ion-beam scanning electron microscopy provide unprecedented, three-dimensional views of cardiac ultrastructures within sample volumes ranging from hundreds of nanometres to hundreds of micrometres. The datasets from these samples are typically large, with file sizes ranging from gigabytes to terabytes and the number of image slices within the three-dimensional stack in the hundreds. A significant bottleneck with these large datasets is the time taken to extract and statistically analyse three-dimensional changes in cardiac ultrastructures. This is because of the inherently low contrast and the significant amount of structural detail that is present in EM images. These datasets often require manual annotation, which needs substantial person-hours and may result in only partial segmentation that makes quantitative analysis of the three-dimensional volumes infeasible. We present CardioVinci, a deep learning workflow to automatically segment and statistically quantify the morphologies and spatial assembly of mitochondria, myofibrils and Z-discs with minimal manual annotation. The workflow encodes a probabilistic model of the three-dimensional cardiomyocyte using a generative adversarial network. This generative model can be used to create new models of cardiomyocyte architecture that reflect variations in morphologies and cell architecture found in EM datasets.

This article is part of the theme issue ‘The cardiomyocyte: new revelations on the interplay between architecture and function in growth, health, and disease’.

## 1. Introduction

Three-dimensional electron microscopy (EM) methods such as electron tomography, focused ion-beam scanning electron microscopy (FIB-SEM) and serial block-face SEM (SBF-SEM) have provided unprecedented three-dimensional views of the cardiomyocyte. We now appreciate that mitochondria form three-dimensional networks that maintain cardiac energy supply [1]. Electron tomography analysis of the cardiac dyad has revealed intricate details about the three-dimensional spatial relationship between t-tubules and the sarcoplasmic reticulum [2]. Two key bottlenecks in analysing three-dimensional EM datasets are image segmentation and three-dimensional morphometric analyses of the segmented datasets. Three-dimensional EM techniques generate data files of the order of several hundred gigabytes to terabytes. These files contain hundreds of image slices that assemble into the three-dimensional volume image stack. The inherent low contrast in EM images makes automated segmentation extremely challenging. A major drawback of traditional image processing methods [3,4] is that the raw images need to be pre-processed carefully to ensure the image processing algorithm can pick up key features of interest. Such pre-processing algorithms could involve simple filtering techniques to deal with the noise, geometric or intensity distribution transformations. Therefore, image segmentation is often conducted manually, which needs substantial person-hours that often result in only a fraction of the data available being processed and analysed.

Segmenting three-dimensional EM stacks is an arduous and tedious task since the texture and intensity variations across all parts of the images are so similar [5]. Manual segmentation is widely used for these datasets, and several tools have been developed with such an approach in mind. However, annotating EM stacks using these tools is time-consuming and laborious, particularly if the three-dimensional volume comprises many image slices. A variety of machine learning approaches have been proposed to address this problem, including ilastik [6], TrakEM2 [7] and Microscopy Image Browser [8]. Although such tools demonstrate acceptable performances, these tools still depend on feature extraction and feature selection and do not capture the optimal features automatically. However, deep learning (DL) automates feature extraction and selection, automatically adapting the underlying parameters (features) to the data [9].

DL has shown unprecedented performance in a wide range of data analysis tasks, including image analysis. Neural networks represent parametric nonlinear models by using nonlinear components of linear combinations of the inputs. These differentiable nonlinear functions are called activation functions which form the infrastructure for nonlinear optimization of such networks. Neural networks are stacks of components called ‘hidden layers' composed of linear mappings of the inputs and nonlinear activation functions. Stacking more hidden layers between the input and output of the network will result in neural network architectures called deep neural networks (DNNs) [10]. Such models start from input data and progressively move towards high-level features using nonlinear mappings. DNNs require relatively large datasets as their topology gets much deeper; hence, they facilitate learning very complex dependencies and intricate structures in data [11].

DL has improved the efficiency of segmenting EM data by minimizing the time required for segmenting such datasets. DNNs are typically used for feature extraction or microscopy image classification. A key advantage over traditional image processing methods is that DNNs automatically extract high-level features for accurate segmentation from the annotated training data. This removes the need for manual fine-tuning of parameters like threshold values that is typical of traditional image processing methods. We have previously applied and benchmarked several existing and customized DNNs to segment mitochondria in EM data [12,13]. We have also previously used traditional image processing approaches to segment mitochondria, as well as Z-discs, myofibrils, cell nuclei. To date, besides the segmentation of mitochondria with neural networks, there are no DL methods to segment multiple structures from cardiac EM data [3,4,14]. In this study, we present a U-net [15] framework to perform the segmentation of mitochondria and myofibrils and Z-discs.

U-net [15] was proposed in 2015 for medical and biological image segmentation. The U-net architecture uses two-dimensional max-pooling layers to downsample the input image data while capturing high-level features, which also benefits computational demand. Moreover, two-dimensional UpSampling layers are used to retrieve the original resolution from high-level features. The network uses two symmetric paths called contractive (encoder) and expansive (decoder) paths to enhance capturing context and localization. In addition to the above, each block in the encoder is connected to the corresponding block in the decoder using skip connections (merging), which boosts the gradient flow in the network during optimization while enhancing localization. As shown in the electronic supplementary material, figure S1, U-net comprises nine blocks in total. Four blocks for each encoder and decoder, and one bottleneck (fifth block). We have modified the topology of the bottleneck for the original U-net by adapting a densely connected bottleneck.

Beyond segmentation, image quantification is an essential task in structural biology investigations. The conventional workflow to obtain statistical descriptors of cell compartments is first to segment the images into categories of interest and then use those masks or labels to generate the three-dimensional structure of the cell or obtain three-dimensional shape and geometrical statistics. This imposes many challenges and costs, including collecting large data samples, annotating, segmenting, and quantifying all available samples. Generative adversarial networks (GANs) are a class of DL models that can learn complex latent features from a dataset in an unsupervised manner. GANs enable us to learn the statistical distributions from images, which removes the bottleneck of manually quantifying image features after segmentation. Several studies have used GANs to extract unsupervised feature representations across different cell types [16–21]. These studies have mainly been applied to two-dimensional images or have only generated relatively low-resolution synthetic images of the order of 256 × 256 pixels. In this study, we present a novel GAN-based generative modelling approach that allows us to map cardiac ultrastructural image data directly into statistical distributions.

We have used a GAN called StyleGAN to create generative models of cardiac ultrastructure. StyleGAN [22] was proposed to enhance image synthesis and resolution with an unprecedented scale ranging from 256 × 256 to 1024 × 1024 pixels. StyleGAN was motivated by style transfer literature where injected noise to the network enables automatic, unsupervised separation of high-level attributes from stochastic variation in generated images. One of the novelties of StyleGAN, among many others, is that latent code is not provided to the generator through an input layer. Instead, image synthesis is initiated from a learned 4 × 512 × 512 constant tensor. This study presents a novel approach of using StyleGAN to generate a two-dimensional probability distribution of cardiomyocyte architecture found within the image slices of the three-dimensional EM volume dataset. This two-dimensional probability distribution can then be used to generate new two-dimensional EM images that can be assembled into a three-dimensional volume of GAN generated cardiomyocyte images. We can use these GAN generated images to extract statistical measures of morphology and spatial distribution automatically. We used this approach to create new instances of the three-dimensional architecture of four sarcomeres of a healthy cardiomyocyte.

**Figure 1.**
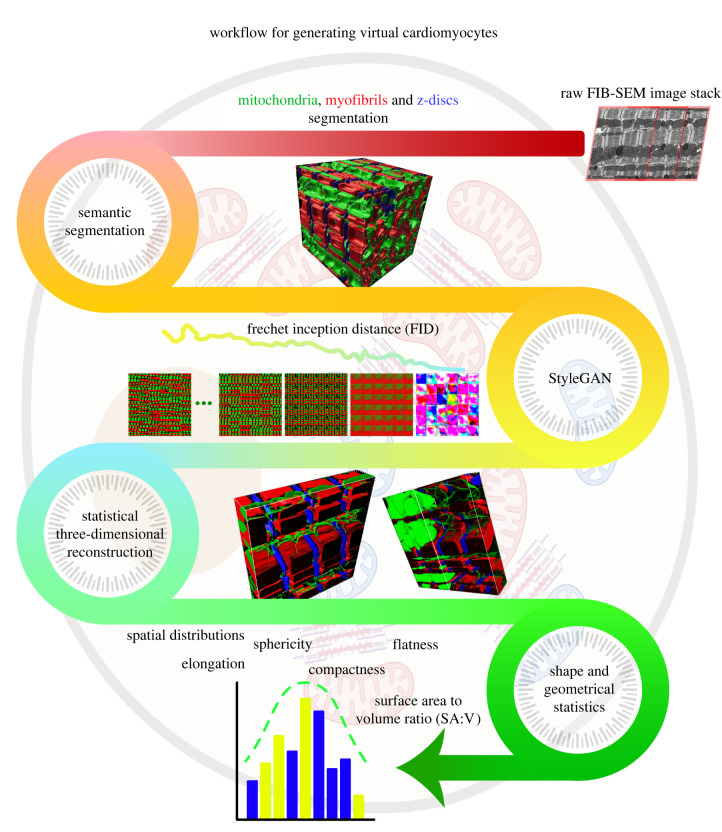
The CardioVinci pipeline for segmentation and generation of the three-dimensional subcellular architecture of cardiomyocytes from electron microscopy data. First, semantic segmentation is used to segment target ultrastructures. Then StyleGAN is used to optimize the error between generator and discriminator. After the images are generated using the trained StyleGAN, three-dimensional statistical representations of cardiomyocytes are reconstructed. Finally, three-dimensional shape and geometric statistics are retrieved using the generated volume.

We have packaged our semantic segmentation U-net and StyleGAN into CardioVinci, a workflow to segment and statistically quantify three-dimensional morphology and organization of mitochondria, myofibrils, and Z-discs. Figure 1 presents the workflow in CardioVinci. To the best of our knowledge, this is the first study to provide a generative model of the three-dimensional ultrastructure of a cardiomyocyte.

## 2. Material and methods

### (a) Workflow

We first provide an overview of the workflow for CardioVinci in this section before giving methodological details. First, mitochondria, myofibrils and Z-discs are segmented using a modified U-net (densely connected bottleneck as shown in figure 1). After the semantic segmentation is performed, these three ultrastructures are segmented with one network through one-time training. Then, the segmented image slices are fed into StyleGAN for the training of the GAN. As shown in figure 1, StyleGAN starts learning very abstract latent space projections. As our monitoring criterion is minimized (here Fréchet inception distance, FID), the latent space projections become more realistic and are more representative of the latent distribution in the actual sample. Upon network convergence, we sort the predicted image slices with the objective of minimizing mutual Jaccard distance. These re-arranged GAN generated image slices form a three-dimensional image stack that can be used to quantify three-dimensional shape and geometric statistics. For this study, we have used compactness, flatness, sphericity, elongation and surface area to volume ratio (SA : V) to quantify three-dimensional statistics.

### (b) Data

We tested our algorithms and workflow on a publicly available FIB-SEM cardiac dataset^1^ [1,12]. We also used CardioVinci on an SBF-SEM dataset of a left ventricular cardiomyocyte extracted from a type 1 diabetic rat tissue sample. Methods for preparing this tissue sample have been described previously [23]. We extracted 30 random 512 × 512 pixel patches from each dataset for training, testing and validation. We manually annotated mitochondria, myofibrils and Z-discs on one slice (2922 × 1166 pixels) FIB-SEM dataset, and then we extracted 30 non-overlapping patches. However, we limited annotations to mitochondria and myofibrils on the SBF-SEM sample after manually annotating two random slices and extracting non-overlapping random patches. We split the annotated and raw images randomly into training, validation and testing by 20/30, 5/30 h and 5/30, respectively. All the random data splits were performed using K-fold cross-validation, and the inference performance is reported based on the best-fold model.

### (c) Training and testing

DNNs are prone to underfitting and overfitting. Such phenomena are described through a vital performance measure called ‘bias-variance tradeoff' [24]. Underfitting occurs when the model fails to capture the underlying pattern owing to high bias and low variance. However, overfitting occurs when the model fits the noise while capturing the corresponding pattern in the data. This results in high variance and low bias. A well-optimized DL model is one that has a low bias and low variance while fitting the training data. Each DNN requires three samples of raw and annotated data for training, validation and testing. We use training data to train the DNN, and intermittently, validation data are used during training to minimize overfitting. To further ensure that the DL model has not been underfitted or overfitted, a separate sample is used for testing the network performance. As highlighted above, we have used approximately 70%, 15%, 15% of the data for training, validation and testing, respectively.

This study implemented all the experiments using DL AMI (Amazon Linux 2) version 46.0 using Amazon Web Services. These experiments were performed on graphical processing units (GPU) instances. We used ‘g4dn.12xlarge' (4 GPUs of a total of 64 GiB memory) and ‘g4dn.metal’ (8 GPUs of a total of 128 GiB memory) for segmentation and training the GAN, respectively. We used semantic segmentation to segment mitochondria, myofibrils and Z-discs simultaneously.

### (d) Semantic segmentation

We modified U-net [15] to segment these ultrastructures using a SoftMax layer consisting of four channels. The SoftMax function is the logistic function generalized to multiple dimensions, enabling multi-class classification. It maps the logits into class probabilities by normalizing the exponents of each logit. The electronic supplementary material, figure S1 shows the architecture of the network. The modified network comprises an input layer of 512 × 512 and 64 feature channels for each layer in the first block. We have used two-dimensional max-pooling with the size and strides of 2 × 2 for blocks 2, 3 and 4 in the network, followed by doubling the feature channels to maximize the localization. Max-pooling layers are used to pass forward the maximum value within a set of activations. They extract low-level features, including edges and expand the network receptive field at no computational cost [11]. We have mainly used rectified linear units as the activation function across the network except for bottleneck and output node. As shown, we have modified the bottleneck by using densely connected convolutional layers, where we have used trainable linear units (TLUs) [13] as the activation function. TLUs boost the convolutional layers' learning capacity and minimize the risk of vanishing gradients during the optimization. We have used densely connected convolutions owing to three reasons [25]: dense connections enable the network to learn a wide range of features and boost the learning capacity of the network by maximizing information flow in the network; moreover, using the previously learned feature maps avoids the risk of very large or near zero gradients, which hinder network optimization [26]; and additionally, dense connections boost gradients flow during the backpropagation.

Once we reach the bottleneck, we have extracted high-level features for segmentation purposes; however, our target variables are also the same shape as the input. Hence, we used two-dimensional upsampling layers and reduced the number of feature channels by half for each of the layers in the consecutive blocks of the network. Upsampling layers increase the feature channels resolution, and convolutional layers maximize the localization. As discussed earlier, we have used the merging layers between the opposite blocks of the encoder and the decoder. Finally, the SoftMax layer is used as the output node with four channels corresponding to mitochondria, myofibrils, Z-discs and the background. It is defined as $p_{n}(x)=\;e^{a_{n}(x)}/\sum_{n^{\prime }=1}^{N}e^{a_{n^{\prime }}(x)}\;$where $a_{n}(x)$ represents the activation in layer *n* at the pixel x ∈ Φ with $\Phi \subset \;Z^{2}$ and *N* is the number of classes. Details regarding the methods used for training, testing and validation are presented in the electronic supplementary material, text.

### (e) Modified StyleGAN for generative modelling

GANs encode the probability distribution of the morphology and spatial organization of image content that they are trained on. CardioVinci uses a GAN to encode the statistical variation in cardiomyocyte architecture observed in semantic segmentation of three-dimensional EM images. This encoded probabilistic model of cardiomyocyte architecture can be used to extract morphological metrics and spatial distributions of mitochondria, myofibrils and Z-discs. The statistical model can also be used to create instances of spatially detailed computational models of the cardiomyocytes that reflect the statistical variation found in real data. We used StyleGAN [22] in this study to extract two-dimensional probability distributions of the organization and morphologies of mitochondria, myofibrils and Z-discs from the semantic segmentation output. Despite its two-dimensional architecture, we were able to map the correlation in cell architecture found between slices in the image stacks into the latent space after a few modifications to the training workflow.

The electronic supplementary material, figure S2 represents the architecture of the used StyleGAN. In a nutshell, StyleGAN comprises a generator and a discriminator in its architecture. As discussed earlier, StyleGAN is different because it uses a two-stage latent modelling mechanism and adopts a new approach to measuring the disentanglement of the latent space (interpolations). Like many other GANs, StyleGAN uses a generator to render an output similar to unseen ground-truth data, which in this case are semantically segmented SEM images of cardiomyocytes. The generator starts updating its gradients from a latent space tensor, which is initially random noise. Then the outputs of the generator (fake samples) are passed to the discriminator along with the real sample. Finally, based on an objective function, the weights for both generator and discriminator are optimized until the generator starts producing fake samples that the discriminator cannot differentiate between fake and real samples.

The StyleGAN was first trained on semantic segmentation outputs generated from the three-dimensional EM dataset. Owing to the TFRecord formatting, we can store a set of features along with the image itself in the TFRecord data. We used one hot-encoded formatting to assign a label to two consecutive image slices as part of building our TFRecord data for training the StyleGAN using segmented image slices. Then we added another input node to the StyleGAN as an embedding layer to embed the correlation across different slices. We followed exactly the same strategy to optimize StyleGAN as highlighted in [22], except we did not employ shuffling during the training. The network convergence was based on FID.

**Figure 2.**
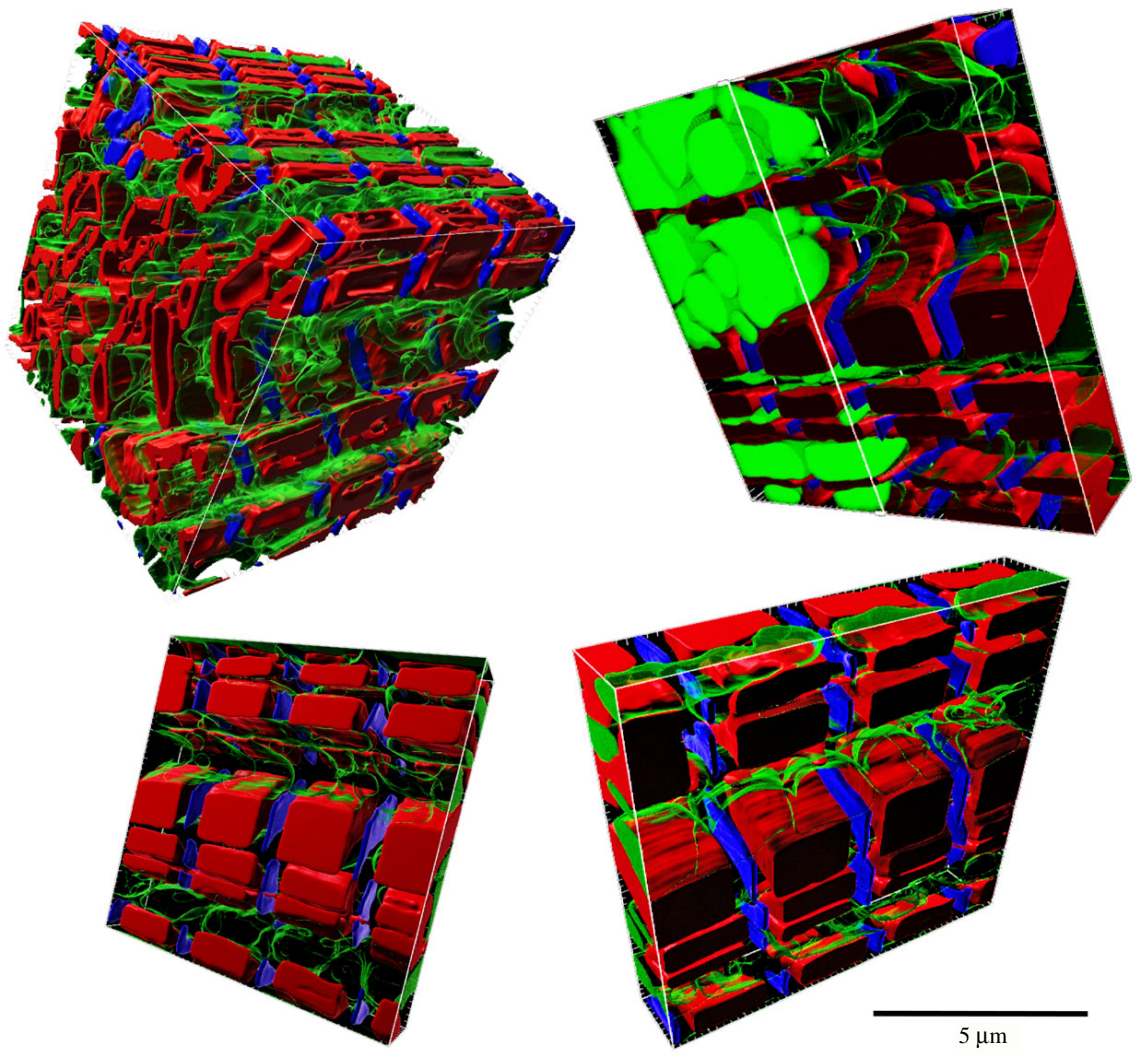
Results of the volume reconstructions from the GAN outputs. Red, blue and green represent the myofibrils, Z-discs and mitochondria, respectively. All the volumes represent approximately four sarcomeres of the cardiomyocytes that have been generated using our trained GAN. The reconstructed volumes are limited to 10 µm × 10 µm × 10 µm (top left) and 10 µm × 10 µm × 2.5 µm.

The trained GAN was then used to generate new two-dimensional images that sampled the encoded probability distribution within the GAN. We then ordered the resulting images into a three-dimensional volume image stack that minimized the Jaccard distance between any two consecutive image slices. This newly assembled three-dimensional image stack is then a generated model of the three-dimensional cardiomyocyte architecture. With the current implementation in CardioVinci, the generated volumes range from 10 µm × 10 µm × 2.5 µm to 10 µm × 10 µm × 10 µm (depending on the number of the random seeds during GAN-based image generation). This volume size spans four sarcomeres of the cardiomyocyte, as shown in figure 2.

## 3. Results

### (a) Semantic segmentation improves feasibility and accuracy

**Figure 3.**
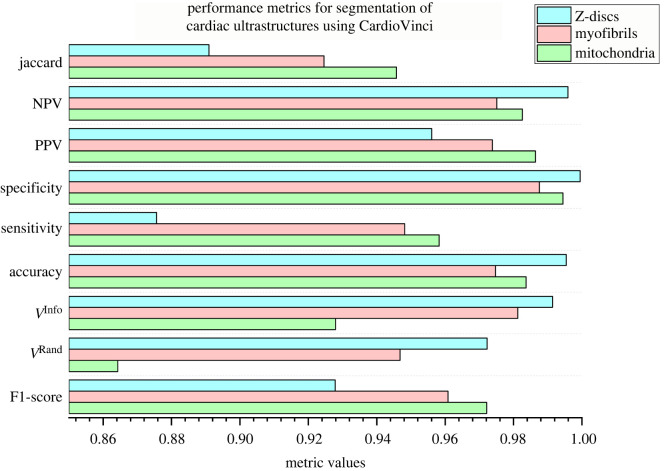
Performance metrics for the segmentation results based on test data. Z-discs have achieved the maximum $V_{\left(\mathrm{thinned}\right)}^{\mathrm{Rand}}$, $V_{\left(\mathrm{thinned}\right)}^{\mathrm{Info}}$, accuracy, specificity and NPV scores compared to mitochondria and myofibrils. PPV and NPV represent positive predictive and negative predictive values, respectively.

Figure 3 represents evaluation metrics for mitochondria, myofibrils and Z-discs on the test sample. Segmenting Z-discs can be challenging using machine learning or DL methods, as discussed in [4,14]. The main challenge is the relatively large label imbalance that makes segmentation of the Z-discs inaccurate. For example, a rather significant label imbalance leads to poor optimization performance when aiming to segment Z-discs only (binary segmentation). However, figure 3 shows that segmenting Z-discs along with mitochondria and myofibrils not only boosts the feasibility of Z-discs segmentation using DL methods but also leads to higher test data performance across various metrics, including $V_{\left(\mathrm{thinned}\right)}^{\mathrm{Rand}}$ and $V_{\left(\mathrm{thinned}\right)}^{\mathrm{Info}}$. These performance measures are explained in more detail in the supplementary material. This result highlights the importance of addressing class imbalance within image volumes. When only segmenting Z-discs, less than 1% of the pixels would be categorized into the positive class (Z-discs in this case), but more than 99% of the pixels would be assigned to the negative class. This imbalance biases neural networks to learn the dominant class pattern [27]. By simply introducing more segmentation categories to the network, we reduce the significant bias in the class distribution of pixels, which leads to better learning and segmentation performance for small structures like Z-discs [27].

### (b) CardioVinci generates realistic models of cardiomyocyte architecture

**Figure 4.**
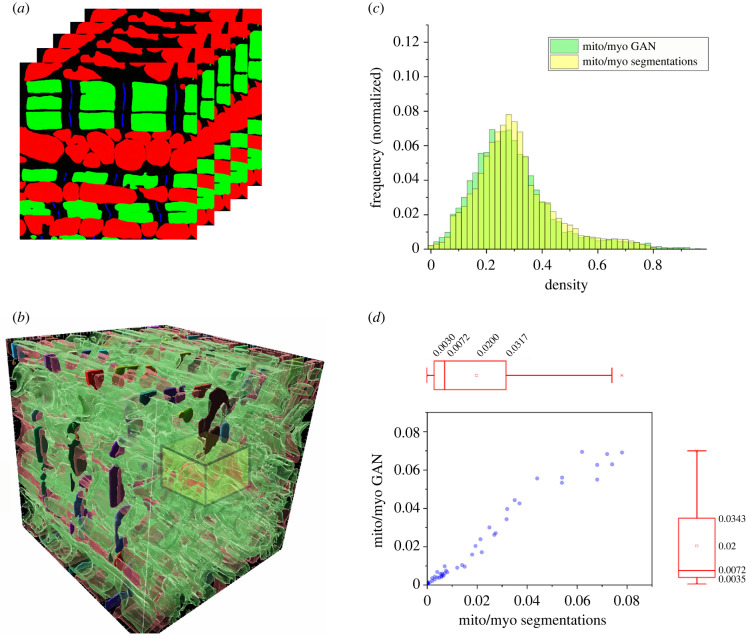
(*a*) Sample two-dimensional slices generated using GAN; (*b*) we have used a sliding kernel 4 µm × 4 µm 4 µm with strides of 0.4 µm on GAN and segmentation volumes to quantify the mitochondria/myofibrils (mito/myo) density; (*c*) normalized frequency distributions for mito/myo density using segmentation and GAN results; (*d*) marginal distribution and corresponding box charts for mito/myo density based on segmentation and GAN volumes.

After semantic segmentation, CardioVinci's StyleGAN assembles a generative model of cardiomyocyte architecture. Figure 4*a* shows a two-dimensional sample image generated by the trained GAN. Figure 4*b* presents a three-dimensional rendering of a generated cardiomyocyte model. We extracted a three-dimensional distribution of the relative density of mitochondria voxels to myofibrillar voxels (mito/myo) within the GAN and semantic segmentation outputs. We passed a 4 µm × 4 µm 4 µm mito/myo density kernel with strides of 0.4 µm across the two three-dimensional volumes. Figure 4*c,d* shows the generated volumes' frequency distributions and marginal boxplots. These plots show that the GAN generated model reflects the spatial distributions of mitochondria and myofibrillar voxels found within the semantic segmentation output.

**Figure 5.**
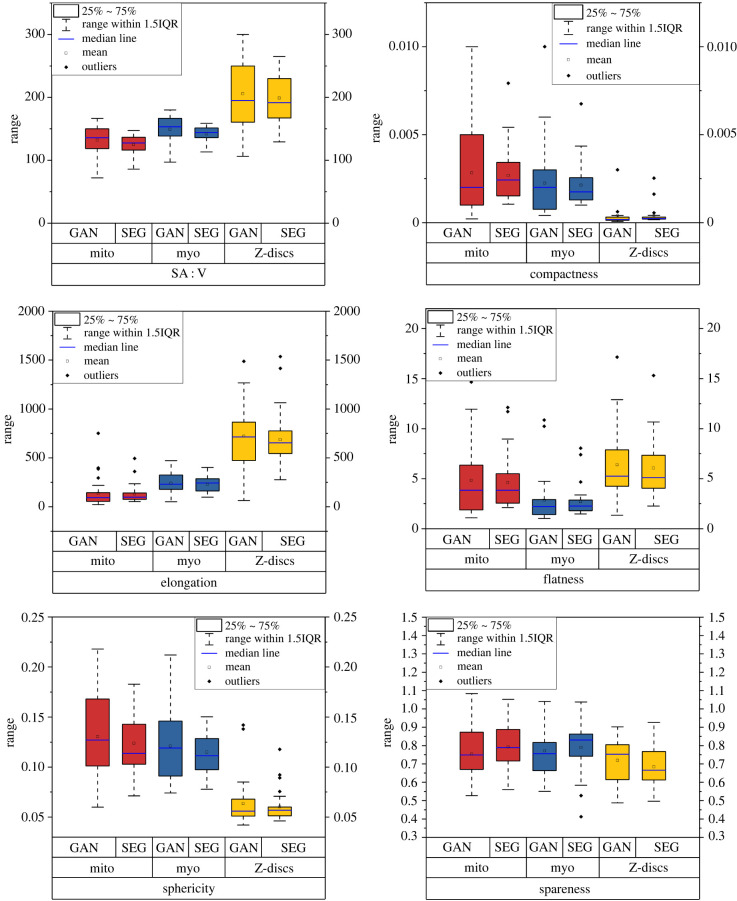
Statistical distribution of surface area to volume ratio (SA : V) and other three-dimensional shape metrics, including elongation, compactness, flatness, spareness and sphericity. These statistics are drawn based on the GAN results and original segmented dataset (SEG).

Figure 5 shows morphometric analyses of the different components of the cardiomyocyte model. These analyses were conducted using a three-dimensional shape analysis plugin [28] for the image processing package Fiji [29]. We compared the statistical distribution of the morphologies of the Z-discs, mitochondria and myofibrils between the GAN generated model and the three-dimensional cardiomyocyte masks output by the semantic segmentation outputs. Figure 5 shows that the distributions generated by the GAN are similar to the distributions from the semantic segmentation dataset. Figure 5 and figure 4*c*,*d* demonstrate the ability of CardioVinci to generate realistic three-dimensional cardiomyocyte models. Moreover, the electronic supplementary material, table S1 represents the one-way ANOVA test results for comparing the means and variances between the ground-truth volume and the sample generated using GAN. The results show that means and variances for the majority of the three-dimensional shape statistics are not significantly different between these samples.

### (c) CardioVinci can be applied to different samples to perform three-dimensional comparative statistical analyses

**Figure 6.**
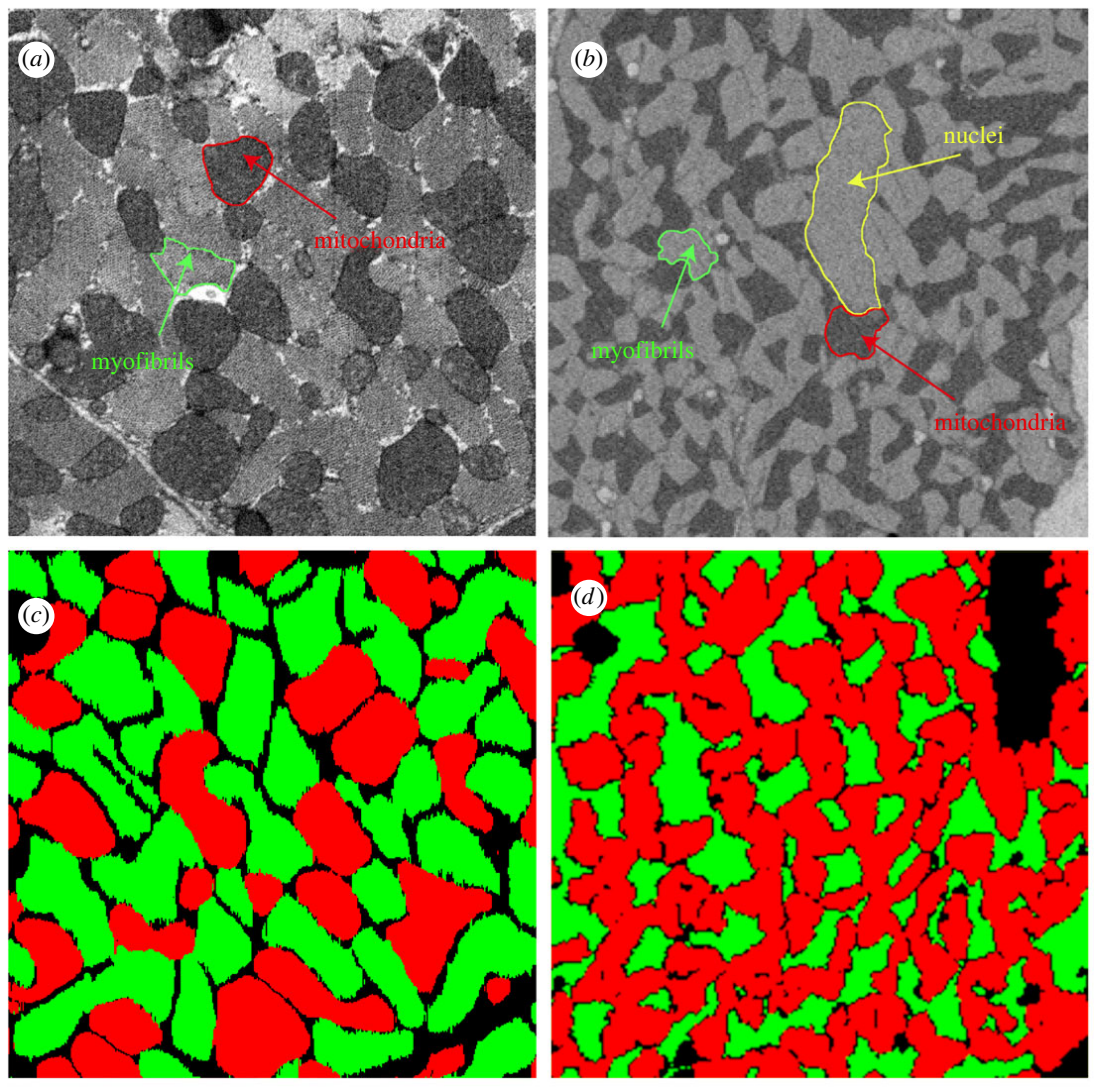
Sample cross-sectional views from GAN reconstructions and raw EM image data. (*a*) Cross-sectional view of the FIB-SEM control sample. (*b*) Cross-sectional view of the SBF-SEM diabetic sample. (*c*) Cross-sectional view of the volume generated using GAN for control sample. (*d*) Cross-sectional view of the volume generated using GAN for diabetic sample. Red and green correspond to mitochondria and myofibrils, respectively.

To demonstrate CardioVinci's use as a pipeline for extracting and comparing statistical distributions within three-dimensional EM datasets, we used CardioVinci to process a three-dimensional SBF-SEM dataset of a left ventricular cardiomyocyte within a sample of rat cardiac tissue with streptozotocin-induced diabetes. Figure 6 provides qualitative comparisons of CardioVinci generated images after training on the FIB-SEM (left column) and SBF-SEM (right column) datasets. Quantitative comparisons were not made owing to three reasons: the two datasets were acquired for completely different experimental studies and laboratories; they were acquired at different spatial resolutions; and the datasets only represent one of healthy control and diabetes-induced heart disease, making it insufficient for any robust statistical comparison.

Comparison of the raw images with their corresponding generated images shows remarkable similarities. For example, we have previously shown using two-dimensional EM images that mitochondria form larger clusters in cardiomyocytes of animals affected by streptozotocin-induced diabetes [23]. This characteristic increased clustering is reflected in the raw image of the three-dimensional SBF-SEM dataset as well as the GAN image in figure 6. The SBF-SEM image volume contains a nucleus that we did not segment, and therefore, the semantic segmentation output contains a void in the nucleus region. CardioVinci's GAN has generated a similar void in the new image in figure 6.

## 4. Discussion and conclusion

This paper proposed CardioVinci as a DL-based pipeline for semantic segmentation and extraction of morphological and spatial statistics properties of cardiomyocytes from three-dimensional EM data. CardioVinci uses a modified U-net for semantic segmentation and a generative adversarial network for extracting statistical distributions from three-dimensional EM data. The generative modelling approach presented in this study is a technical breakthrough. Although many studies have applied GANs to microscopy images before [16–18,20,30–34], most of these studies only analyse two-dimensional images and typically generate images of very small pixel resolution (approx. 256 × 256 pixels). Moreover, there are very limited studies that use generative modelling to quantify cellular morphology and localization. In one study [16], the authors proposed using an adversarial autoencoder to capture nuclear and other ultrastructural variations using two-dimensional segmented confocal data. Variational autoencoders (VAEs) learn the underlying variations in data through adapting latent space. The limitation of the disentanglements in the linear subspaces of latent space for VAEs limits the interpolation of the latent vector, hence limiting nonlinear changes in the images [22]. The authors extended the same study by using 128 × 96 × 64 cubic voxels confocal data to integrate resulting ultrastructures of human-induced pluripotent stem cells in three dimensions. This study uses two autoencoders to optimize two different latent vectors, making the training infeasible for high-resolution images such as FIB-SEM data. In another study [20], the authors have used the same two-dimensional confocal image data from the previous study [16] and proposed modifying conditional GANs using newly proposed skip connections. The authors have shown that the proposed method outperforms the previous study based on the log-likelihood measure by maximizing the localization. However, this study is limited to two dimensions and performs the latent space projection using only mutual nuclear shape and individual subcellular proteins (one versus the rest).

In this paper, we have trained the two-dimensional StyleGAN to generate three-dimensional models of cardiomyocytes using a novel methodology that has not been applied before. To the best of our knowledge, our approach is the first computationally efficient method to assemble generative models of any three-dimensional EM data. StyleGAN not only provides an efficient architecture for training high-resolution images up to 1024 × 1024 pixels but also facilitates highly nonlinear latent space interpolations owing to two-stage latent variable modelling. Moreover, adding noise to the generator after each convolution separates high-level features (subcellular ultrastructures) from stochastic variation (morphology). In addition to the above, we have proposed to train StyleGAN using three ultrastructures simultaneously, which circumvents the challenge of additional steps for subcellular integration. In addition to the above, StyleGAN uses perceptual path length to ensure that latent space is disentangled. This is a major breakthrough from the previous studies as the disentanglement studies were ill-posed owing to unknown variation factors [22]. StyleGAN uses perceptually based pairwise image distance, which is calculated as a weighted difference between two VGG16 embeddings. The weights are optimized to resemble the human perceptual similarity judgements leading to fine high-resolution latent space projections.

GANs can be directly trained on raw data; however, they require many more images than the number we used in this study to comprehensively capture the complex spatial properties in the images. Indeed, our experience was that the low contrast in EM images compounds the need for more training data. We have circumvented this perennial issue in DL by performing an intermediary segmentation step to reduce data complexity. We have applied semantic segmentation as an intermediary step towards the creation of a generative model. The StyleGAN was trained on segmentation masks generated by semantic segmentation. Our results in figures 4–6 show that this practical approach is successful in generating realistic cardiomyocyte models from three-dimensional EM data. Although we have only segmented three components in this study, CardioVinci can easily be scaled to extract segmentations and statistics of other components, including t-tubules, sarcoplasmic reticulum and nuclei as well. Indeed, figure 6 demonstrates that the GAN learned to generate a region of black voxels corresponding to the nucleus that was not annotated for semantic segmentation. Moreover, owing to highly nonlinear latent space interpolations, one can generate a wide range of latent space projections, leading to a handful of statistical cellular representations. The electronic supplementary material, table S2 shows results from the statistical comparison of the three-dimensional shape metrics between 25 generated volumes using CardioVinci (each having 96 × 512 × 512 cubic voxels). As shown, CardioVinci can generate consistently statistically similar volumes. However, we show in the electronic supplementary material, figure S3 that the generated volumes are structurally different by quantifying the mutual three-dimensional Jaccard distance between the generated volumes. This means that one could generate as many mutually structurally exclusive but statistically similar geometries (three-dimensional shape statistics such as compactness, flatness, elongation, etc.). However, the training sample size limits the number of mutually exclusive volumes [22].

Despite its successful application in quantifying three-dimensional statistics of cardiomyocytes, we found a few challenges when using CardioVinci. First of all, it requires encoding the consecutive slices in TFRecord as a preliminary data prep step. We understand that this could be challenging when large volumes are used; such encodings should be handled with care owing to missing or noisy image slices. Secondly, performing a grid search to sort and reconstruct the generated slices in three dimensions could hinder the efficiency of automating three-dimensional sample reconstructions and quantifications. This could be particularly challenging when a large number of samples are used. In CardioVinci, we optimize the Jaccard distance between the generated images by minimizing it between consecutive image slices. Once the optimization is performed, we simply order the generated data to obtain the statistical three-dimensional volume. Such a task could be cumbersome in the presence of large tissue samples. Finally, owing to highly intricate structures in three-dimensional EM data, CardioVinci requires a user to segment the data as a preliminary step before training the StyleGAN. Indeed, this is equivalent to the labelling step in the previous studies. However, our experience with SEM data segmentation has shown that segmenting such volumes are viable with performing only one or two image slices.

CardioVinci presents an exciting opportunity for the research community to generate their own probabilistic models of different aspects of components of cardiomyocyte architecture like dyads or mitochondria networks. We have developed two generative models, each trained on one FIB-SEM and one SBF-SEM dataset that represent a healthy and diabetes affected cardiomyocyte, respectively. A more comprehensive generative model can be easily generated by including more image patches across a larger available dataset. We have made CardioVinci publicly available to promote community engagement in this regard.

CardioVinci is a scalable pipeline that can generate statistical representations of three-dimensional EM data of cardiomyocytes given the ultrastructures of interest. Our future aim is to use the style mixing capability of the StyleGAN to generate synthesized variations across different tissue blocks or even tissue types. We encourage the research community to train CardioVinci's generative model to their own datasets for time-efficient three-dimensional EM studies.

## Data Availability

All codes and GAN outputs have been made available via GitHub. The data are provided in the electronic supplementary material [35].

## Endnote

^1^
https://labalaban.nhlbi.nih.gov/files/HeartDatasetS1.tif.
